# Transcriptome Analysis of Brain and Skin Reveals Immune Responses to Acute Hypoxia and Reoxygenation in *Pseudobagrus ussuriensis*

**DOI:** 10.3390/ani14020246

**Published:** 2024-01-12

**Authors:** Qing Liu, Yuxing Li, Yang Cao, Libo Gu, Tongyao Li, Yu Liu, Jing Song, Weiwei Wang, Xianzong Wang, Bugao Li, Shaozhen Liu

**Affiliations:** 1 College of Animal Science, Shanxi Agricultural University, Jinzhong 030800, China; liuqing_sxau@126.com (Q.L.); yuxingli1999@163.com (Y.L.); caoyang0926@126.com (Y.C.); 18313546597@163.com (L.G.); dayaoayao@163.com (T.L.); 18342255015@163.com (Y.L.); songjingoak@163.com (J.S.); sxndweiwei@163.com (W.W.); xianzong_wang@126.com (X.W.); 2 Shanxi Key Laboratory of Animal Genetics Resource Utilization and Breeding, Jinzhong 030800, China

**Keywords:** *Pseudobagrus ussuriensis*, hypoxia, skin, brain

## Abstract

**Simple Summary:**

*Pseudobagrus ussuriensis* is a high-value fish species widely distributed in various river basins across China. Its benthic lifestyle and lack of scales make its skin susceptible to damage, leading to diseases and mortality. Hypoxia represents a significant factor affecting fish health, whether in captive environments or natural water bodies. Exposure to low oxygen levels can result in various adverse effects, including causing an immune response in fish. In this study, we aimed to elucidate the differential immune responses of distinct tissues to acute hypoxia and reoxygenation by analyzing the histophysiological and transcriptional changes in the brain and skin of *P. ussuriensis*. Our findings revealed that both the brain and skin exhibited immune responses to hypoxia, which persisted even after reoxygenation. However, it was observed that the overall immune response intensity in the brain was lower than that in the skin. This research provides novel insights into the molecular mechanisms underlying the response of *P. ussuriensis* to hypoxia stress.

**Abstract:**

*Pseudobagrus ussuriensis* is an unscaled fish that is more susceptible to skin damage than scaled fish. To investigate the impacts of hypoxia and reoxygenation on skin and brain immunity, juvenile *P. ussuriensis* were subjected to hypoxia conditions (DO: 0.8 ± 0.05 mg/L) for durations of 0, 3, 6, and 12 h, followed by 12 h of reoxygenation (DO > 6 mg/L). Histological analysis showed a significant increase in the number of skin mucosal cells after 12 h of hypoxia and a significant decrease after 12 h of reoxygenation when compared to the control group. As the duration of hypoxia increased, an increase in antioxidant (SOD, CAT, GSH, MDA) and immune (cortisol, LZM) physiological parameters of the skin and brain appeared. The results of transcriptomic studies showed that the number of differential genes was greater in skin than in brain. Most of the immune pathways in both tissues under hypoxia conditions were all nonspecific immunity (TNF, IL-17, chemokines), while both tissues maintained their homeostasis through active energy supply and cell cycle regulation. Meanwhile, both physiological parameters and RNA transcriptome results showed that 12 h of reoxygenation could not completely eliminate the negative effects of 12 h of hypoxia. This study offers new insights into the immune responses of *P. ussuriensis* skin and brain during acute hypoxia and reoxygenation.

## 1. Introduction

Oxygen is indispensable for the survival of higher organisms. Dissolved oxygen concentrations in water can undergo diverse and complex changes influenced by physical, chemical, and biological factors. Organisms in aquatic environments are more susceptible to fluctuations in oxygen levels than their terrestrial counterparts. Due to the influence of factors such as global warming, dissolved oxygen levels in many freshwater lakes are steadily decreasing. This presents a significant threat to the stability of freshwater ecosystems and the survival of the various fish species residing within them. Hypoxia is an adverse factor in fish health in the long term [1].

Exposure to low oxygen levels can result in various oxidative damages and oxidative stress in fish [2], leading to alterations in their behavior, physiological and biochemical indicators, metabolisms, and immune responses [3]. In hypoxia stress tests involving two rockfish species, the escape time required by copper rockfish (*Sebastes caurinus*) progressively increased as the dissolved oxygen levels decreased [4]. During acute and chronic hypoxia stress tests on Nile tilapia (*Oreochromis niloticus*), fish swimming speeds decreased with increases in the duration of stress. Glycolysis is an energy supply pathway under low oxygen. Excessive glycolysis accelerates the production of reactive oxygen species (ROS) and thus causes oxidative damage to the body. Living organisms regulate ROS levels in vivo through antioxidant enzymes and antioxidants (e.g., SOD, CAT, and GSH) to mitigate the damage caused by oxidative stress [5]. MDA, as the end product of lipid peroxidation due to the action of free radicals on lipids, is utilized to assess oxidative damage and cellular injury within an organism [6]. In cases of acute hypoxia stress, it was found that carbohydrates became the primary energy source for these fish, while under chronic hypoxia, lipolysis could replace glycolysis to provide energy to the fish [7]. In the hypoxia stress test on small yellow croaker fish (*Larimichthys polyactis*), liver SOD and CAT activities as well as MDA content gradually increased with increasing stress time [8]. Prolonged exposure to low oxygen conditions was shown to disrupt and disorganize the gut mucosal immune system of Atlantic salmon (*Salmo salar*) [9].

Fish primarily rely on their intestines, gills, and skin as crucial mucosal immune barriers to defend against external pathogen invasions. In comparison to terrestrial organisms, aquatic fish encounter more substantial mucosal immune challenges due to their unique underwater environment. This distinct environmental difference has also resulted in different immune system patterns in fish compared to terrestrial organisms [10]. The skin, as the body’s largest organ, plays a crucial role in safeguarding fish from external disturbances and maintaining their physiological homeostasis [11]. Unscaled fish, due to the absence of protective scales, are more vulnerable to damage that can result in diseases or even fatalities. Studies on the skin histology, physiological and biochemical parameters, and transcriptomes of yellow catfish (*Pelteobagrus fulvidraco*) under acute hypoxia demonstrated that prolonged exposure to hypoxia stress led to tissue damage and reduced immunity in *P. fulvidraco* [12]. Short-term exposure to hypoxia significantly elevated the levels of hypoxia markers such as lactate dehydrogenase (LDH) activity, lactate, and glucose in the skin mucus of blue gourami (*Trichogaster trichopterus*) [13]. The mucus produced on the mucosal surface and the number of mucus cells were also used to assess the health and stress status of the fish. Mucus has been employed as an indicator of fish health in low-oxygen stress tests conducted on rainbow trout [14] and in stress tests involving gilthead seabream (*Sparus aurata* L.) [15]. The gills of juvenile orange-spotted groupers (*Epinephelus coioides*) exposed to suspended sediments exhibited hypoxia responses accompanied by subsequent increases in the number of mucus cells [16].

The brain, being the principal organ of a vertebrate’s central nervous system, regulates the life activities of an organism. Damage or infection to the brain can have numerous adverse effects on various organ systems in fish [17]. Under hypoxia, energy synthesis pathways within the brains of darkbarbel catfish (*Pelteobagrus vachelli*) are inhibited, impacting energy-dependent biological processes [18]. Hypoxia significantly upregulated the expression of genes related to stress immunity, such as *IL*-*1β* and *HSP90*, in the brains of Ya fish (*Schizothorax prenanti*) [19]. In chronic hypoxia tests on *Takifugu rubripes*, active nerve repair and neovascularization-reduced brain damage were observed [20].

*P. ussuriensis* is a significant native fish species in China and is distributed in various water systems across the country. Known for its high-quality meat and substantial aquaculture potential, extensive research has been conducted on its reproductive and nutritional aspects [21,22,23]. The fish’s population in the wild has declined dramatically due to overfishing. In the study of the suffocation point of *P. ussuriensis* of different sizes and at different temperatures, researchers found that as a benthic fish, *P. ussuriensis* has high requirements for dissolved oxygen [24]. And we also found that *P. ussuriensis* was easily subjected to low oxygen and died during our fishing and transporting process. The pectoral and dorsal fins of *P. ussuriensis* have sharp spines, which, together with its lack of scales, makes it easy for them to scratch each other’s skin and cause wound infections during stressful situations. However, there remains a significant gap in our understanding of its responses to acute hypoxia and subsequent immune reactions after reoxygenation. With the maturation and continued advancement of RNA sequencing technology, transcriptome analysis has become a valuable tool in the study of exogenous infections and immunology in fish [25]. Analysis of the skin transcriptome of mud loach (*Misgurnus anguillicaudatus*) has further illuminated the immune function and molecular mechanisms associated with fish skin [26]. In this study, we investigated the histological, physiological, and transcriptomic changes in the skin and brains of *P. ussuriensis* following acute hypoxia and reoxygenation. Our research aimed to provide valuable insights into the adaptive strategies of different tissues in *P. ussuriensis* when facing hypoxia conditions, as well as their recovery mechanisms following reoxygenation.

## 2. Materials and Methods

### 2.1. Experimental Fish Management and Hypoxia Treatment

The *P. ussuriensis* individuals used in the experiment were self-propagated juveniles from our laboratory facility. The breeding stock consisted of parents sourced from the Hutuo River in Shanxi, China. Healthy, disease-free individuals (mean length of 7.73 ± 0.44 cm and mean weight of 8.56 ± 1.22 g) were carefully selected and acclimated for two weeks in a 100-liter aquarium equipped with recirculating filtration systems, maintaining a temperature of 15 °C and dissolved oxygen levels of above 6 mg/L. The aim of this acclimation period was to eliminate potential stress factors related to transport and environmental changes. During the temporary feeding period, the *P. ussuriensis* individuals were fed with commercial pellet feed for yellow catfish (*Tachysurus fulvidraco*) (produced by Tongwei Co., Ltd., Chengdu, China).

In preliminary experiments conducted at a temperature of 15 °C, we observed specific responses in *P. ussuriensis* related to the variations in the dissolved oxygen levels. When dissolved oxygen concentrations approached 1.5 mg/L, an increase in respiration rate was observed in all *P. ussuriensis*. At dissolved oxygen levels of 0.65 mg/L, 90% of *P. ussuriensis* appeared to float. When the dissolved oxygen level reached 0.45 mg/L, all *P. ussuriensis* suffocated and died within one hour. Based on these findings, we selected a dissolved oxygen concentration of 0.8 ± 0.05 mg/L as the acute low-oxygen stress level for our study. In order to maintain this dissolved oxygen concentration, we used hoses with air stones to connect gas tanks with flow meters (one hose to the oxygen tank and the other to the nitrogen tank). The two air stones and an oxygen meter probe were plunged into the aquarium, which was closed with a transparent plastic film, and the flow meters of the two cylinders were adjusted until the dissolved oxygen was maintained at the target concentration. Throughout the experiment, we monitored the water’s dissolved oxygen concentration using a dissolved oxygen meter (AZ8413, Taiwan, China).

The experimental groups were arranged as follows: a control group, C; a 3-hour hypoxia group, N3; a 6-h hypoxia group, N6; and a 12-h hypoxia group, N12. Additionally, a reoxygenation group, O12, was established, where the fish that had undergone 12 h of hypoxia were transferred to a normal dissolved oxygen environment for 12 h.

### 2.2. Sample Collection

After each experimental group had reached its designated treatment time, 15 fish were randomly selected and removed from the aquarium. The fish were anaesthetized with MS-222 and then rapidly dissected to collect the skin (S) from the backs of the fish and their intact brains (B). From these 15 fish, the tissues from 9 individuals (grouped as 3 samples with each containing a mix of tissues) were carefully collected and placed into cryopreservation tubes free of DNase and RNase. These tubes were then rapidly frozen in liquid nitrogen and subsequently stored in a −80 °C freezer for preservation. The mixed tissue samples were designated for transcriptome sequencing and subsequent qPCR analysis. In addition, tissues from three more fish were utilized to analyze their physiological indices, while the skin samples from the remaining three fish were preserved in Born’s solution for fixation, intended for subsequent histological analysis.

### 2.3. Skin Histological Analysis

The finished fixed skin tissue was removed from the fixative and trimmed flat. After gradient alcohol dehydration (75% alcohol for 4 h, 85% alcohol for 2 h, 90% alcohol for 2 h, 95% alcohol for 1 h, anhydrous ethanol I for 30 min, anhydrous ethanol II for 30 min, alcohol benzene for 5–10 min, xylene II for 5–10 min), the tissues were dipped in wax (65 °C melting paraffin I for 1 h, 65 °C melting paraffin II for 1 h, 65 °C melting paraffin III for 1 h). The wax-impregnated tissues were embedded in an embedding machine. The trimmed wax blocks were sliced on a paraffin slicer to a thickness of 4 μm, and the slices were then dewaxed (the sections were placed into environmentally friendly dewaxing transparent liquid Ⅰ for 20 min, environmentally friendly dewaxing transparent liquid Ⅱ for 20 min, anhydrous ethanol Ⅰ for 5 min, and anhydrous ethanol Ⅱ for 5 min—75% alcohol for 5 min, rinsed with tap water) and stained with AB-PAS. The slices were successively inserted into anhydrous ethanol I for 5 min, anhydrous ethanol II for 5 min, anhydrous ethanol III for 5 min, xylene I for 5 min, xylene II for 5 min, and finally sealed with neutral gum. After the staining procedure, we examined the variations in mucous cells under a light microscope and captured them with photographs for further analysis.

### 2.4. Determination of Oxidative Stress and Immunological Indices in the Fish Brains and Skin

We used an enzyme-linked immunosorbent assay (ELISA) kit (Shanghai Enzyme-linked Biotechnology Co., Ltd., Shanghai, China) to measure the activity of superoxide dismutase (SOD) and catalase (CAT) in the skin and brain tissues. Additionally, we determined the levels of cortisol, lysozyme (LZM), malondialdehyde (MDA), and glutathione (GSH) in these tissues. We selected a homogenizing solution of PBS (PH of 7.2–7.4 at a concentration of 0.01 mol/L) with a homogenate proportion of 10%. The homogenization process was performed using a tissue homogenizer, and the supernatant was collected by homogenizing and centrifuging on an ice bath at 5000 r/min for 15 min. The supernatant was used for testing, and we followed the manufacturer’s instructions to measure the various indicators. Each group was tested in triplicate for accuracy and consistency. The procedure is to add 50 μL of the standard to the enzyme-coated plate, seal the plate with a sealing film, and incubate it at 37 °C for 30 min. Dilute the 30-fold concentrated washing solution 30-fold with distilled water and prepare for use. Remove the membrane carefully and wash 5 times. Add 50 μL of enzyme reagent to each well, except the blank wells. The warming and washing steps described above were then repeated. Color development solution was added to develop the color for 10 min at 37 °C, protected from light. The reaction was terminated by adding 50 μL of termination solution per well. The absorbance (OD) of each well was measured sequentially at 450 nm. The standard curve was calculated by the concentration of the standard, and the level of each index was calculated accordingly.

### 2.5. Total RNA Extraction and Transcriptome Sequencing

The transcriptome sequencing and subsequent cDNA library construction were conducted by OE Biotech (Shanghai, China). RNA samples were extracted from the brain and skin of groups C, N12, and O12. Total RNA was extracted using a mirVana miRNA Isolation Kit (Ambion, Austin, TX, USA) following the manufacturer’s protocol. RNA integrity was evaluated using an Agilent 2100 Bioanalyzer (Agilent Technologies, Santa Clara, CA, USA). Samples with RNA integrity number (RIN) ≥ 7 were subjected to subsequent analysis. The libraries were constructed using TruSeq Stranded mRNA LTSample Prep Kit (Illumina, San Diego, CA, USA) according to the manufacturer’s instructions. Then, these libraries were sequenced on the lllumina sequencing platform (HiSeqTM 2500) and 150 bp paired-end reads were generated.

### 2.6. Quality Control and De Novo Assembly

Raw data (raw reads) were processed using Trimmomaticl. Reads containing ploy-N and low-quality reads were removed to obtain clean reads. After removing adaptor and low-quality sequences, the clean reads were assembled into expressed sequence tag clusters (contigs) and de novo assembled into transcript by using Trinity2 (vesion: 2.4) with the paired-end method. The longest transcript was chosen as a unigene based on the similarity and length of a sequence for subsequent analysis.

### 2.7. Functional Annotation

The function of the unigenes was annotated by alignment of the unigenes with the NCBI nonredundant (NR), SwissProt, and Clusters of orthologous groups for eukaryotic complete genomes (KOG) databases using Blastx with a threshold E-value of 10^−5^. The proteins with the highest hits to the unigenes were used to assign functional annotations thereto. Based on the SwissProt annotation, Gene Ontology (GO) classification was performed by the mapping relation between SwissProt and GO terms. The unigenes were mapped to the Kyoto Encyclopedia of Genes and Genomes (KEGG) database to annotate their potential metabolic pathways.

### 2.8. Analysis of Differentially Expressed Unigenes (DEGs), Cluster Analysis, GO and KEGG Enrichment

FPKM and read count values of each unigene were calculated using bowtie2 and eXpress. DEGs were identified using the DESeq (2012) functions estimateSizeFactors and nbinom Test. *p* value 0.05 and foldChange > 2 was set as the threshold for significantly differential expression. Hierarchical cluster analysis of DEGs was performed to explore transcript expression patterns. GO enrichment and KEGG pathway enrichment analysis of DEGs were, respectively, performed using R based on the hypergeometric distribution.

### 2.9. qPCR Validation of the Transcriptome Data

The total RNA was reversed using a PrimeScript™ RT reagent Kit with a gDNA Eraser kit (Perfect Real Time) from Takara Japan. The primers were designed using Premier5 software, and *β*-*Actin* was employed as the reference gene [27]. Primers were synthesized by Sangon Biotech (Shanghai, China) Co., Ltd. The obtained cDNA and primers were added to the reaction system and subjected to real-time fluorescence quantification according to TB Green^®^Premix Ex Taq™II (Tli RNaseH Plus) reagent manufactured by Takara Corporation, Japan. The sequences of each primer are provided in Table 1. The gene expression levels were calculated using the 2^−ΔΔCT^ method [28].

### 2.10. Statistical Analysis

Experimental data were expressed as means ± standard deviation (SD). One-way ANOVA and multiple comparisons were conducted using SPSS 22.0. The physiological indicators and qPCR data were analyzed and graphed using Origin 2021.

## 3. Results

### 3.1. Histological Changes in the Skin

**Figure 1:**
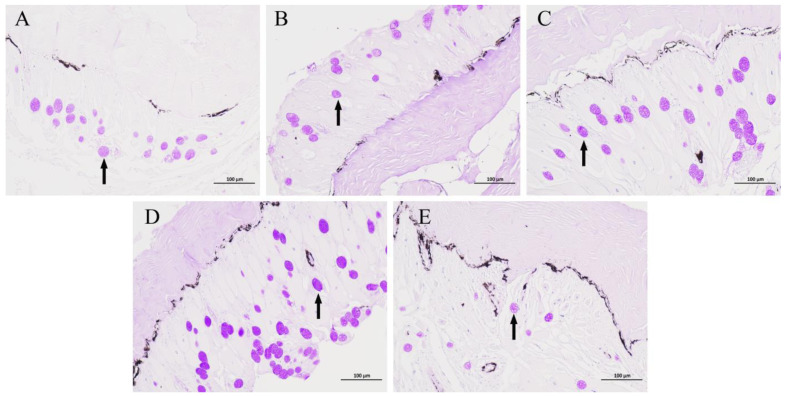
AB-PAS staining of the *P. ussuriensis* skin tissues. Groups C, N3, N6, N12, and O12 correspond to panels (**A**–**E**), respectively. The mucous cells are indicated by black arrows. Scale bar: 100 μm.

The results of the mucus cell staining using the AB-PAS method are depicted in Figure 1. In comparison to group C, groups N3 and N6 showed no significant changes in the number of mucus cells. In contrast, the N12 group showed a significant increase in the number of mucous cells. After reoxygenation, a significant decrease in the number of mucous cells was observed in the O12 group. The mucus cell counts and significance analysis for each group can be found in Table 2.

### 3.2. Antioxidant and Immune Indicators in the Brains and Skin

**Figure 2:**
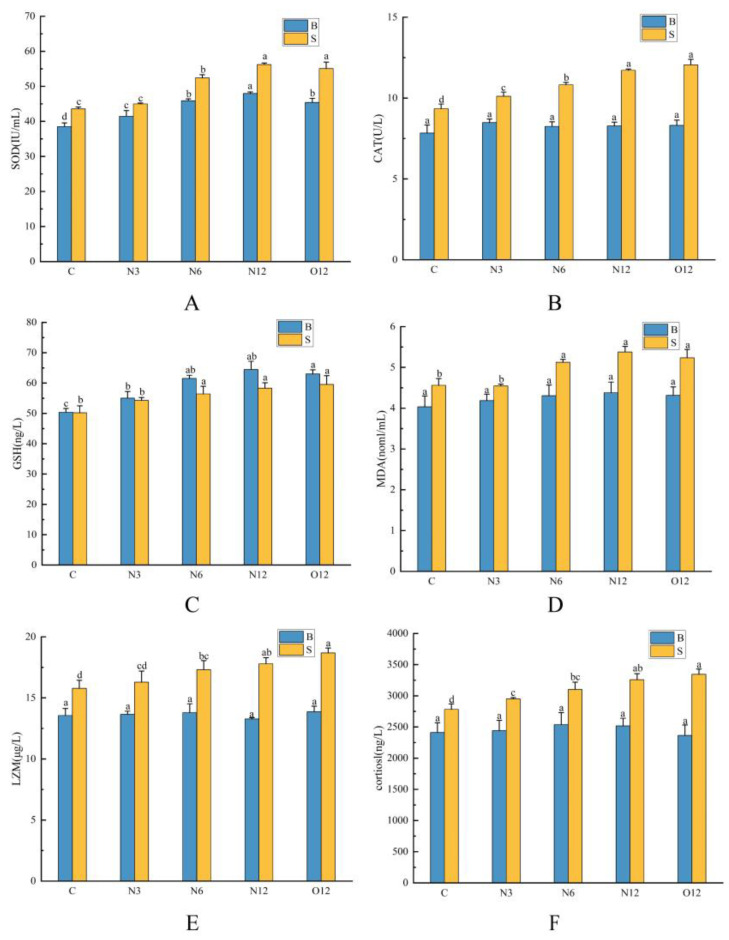
Effects of hypoxia on SOD (**A**), CAT (**B**), GSH (**C**), MDA (**D**), LZM (**E**), and cortisol (**F**) levels in the brain (B) and skin (S) tissues of *P. ussuriensis*. The different lowercase letters indicate significant differences at *p* < 0.05.

As the duration of hypoxia increased, there were significant increases in skin SOD and CAT activities, as well as GSH and MDA contents. In the brain tissues, most of the oxidative stress indicators significantly increased, with the exception of the MDA content and CAT activity, which remained stable. However, the indicators that significantly increased after 12 h of reoxygenation remained significantly elevated compared to group C (Figure 2A–D). The skin LZM and cortisol levels showed significant increases with prolonged exposure to hypoxia, and these elevated levels were maintained even after reoxygenation. In contrast, the cortisol and LZM levels in the brain tissues did not exhibit significant changes, with only a minor initial increase during the stress period before returning to levels similar to those of group C (Figure 2E,F).

### 3.3. Transcriptome Results and Quality of Data

In this experiment, transcriptome sequencing was conducted on 18 samples, resulting in a total of 126.58 G of clean data. The effective data volume for each sample ranged from 6.68 G to 7.42 G. The Q30 base distribution ranged from 93.32% to 94.43%, with an average GC content of 46.72%. A total of 61,825 unigene entries were assembled, with a combined length of 92,552,050 base pairs and an average length of 1497.0 base pairs (Table 3).

### 3.4. Differential Gene Analysis

#### 3.4.1. Differential Gene Analysis of the Skin Samples

**Figure 3:**
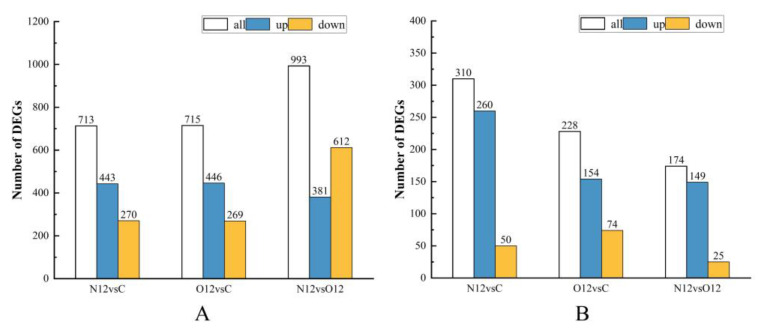
Comparison of differential gene expression in the different groups of brain and skin samples. Bar graphs (**A**) (skin) and (**B**) (brain) show different comparisons of upregulated and downregulated genes.

An analysis of the differential gene results in the different skin groups revealed that in the comparison of group N12 to group C, there were a total of 713 differential genes, with 443 being upregulated and 270 being downregulated. When comparing group O12 to group C, there were 715 differential genes, with 446 being upregulated and 269 being downregulated. In the comparison of group N12 to group O12, a total of 993 differential genes were found, with 381 being upregulated and 612 being downregulated (Figure 3A).

#### 3.4.2. Differential Gene Analysis of the Brain Samples

In analyzing the results of the differential genes in the different brain groups, it was observed that in the comparison between group N12 and group C, there were a total of 310 differential genes, with 260 being upregulated and 50 being downregulated. When comparing group O12 to group C, there were 228 differential genes, with 154 being upregulated and 74 being downregulated. In the comparison between group N12 and group O12, a total of 174 differential genes were found, with 149 being upregulated and 25 being downregulated (Figure 3B).

### 3.5. GO Enrichment Analysis

**Figure 4:**
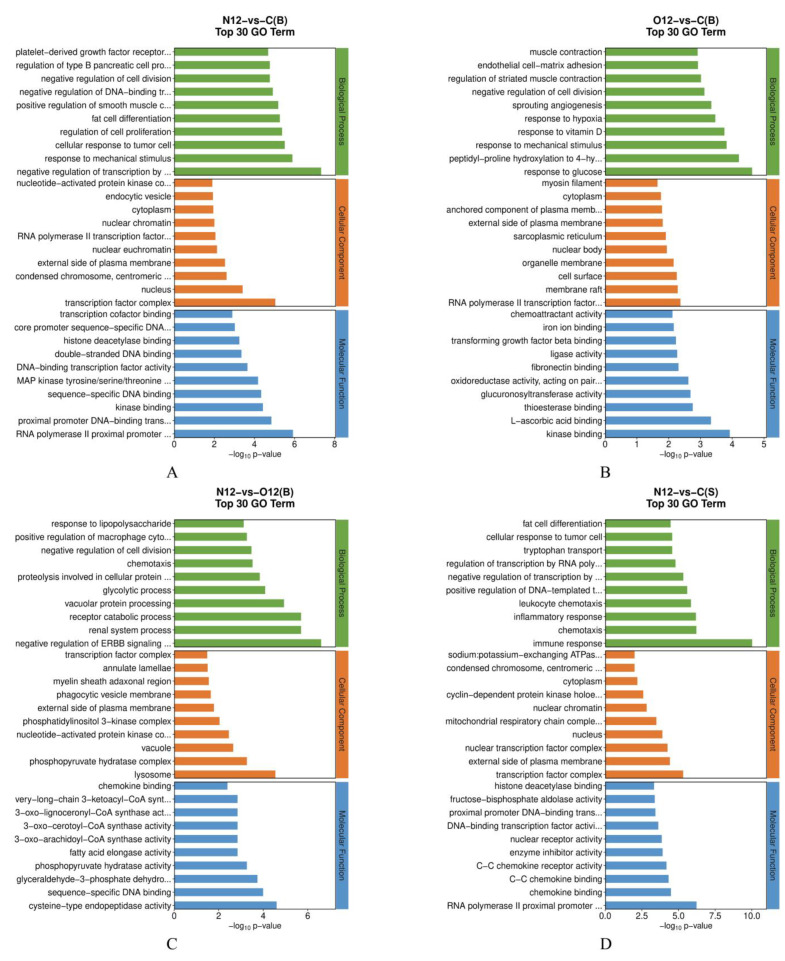

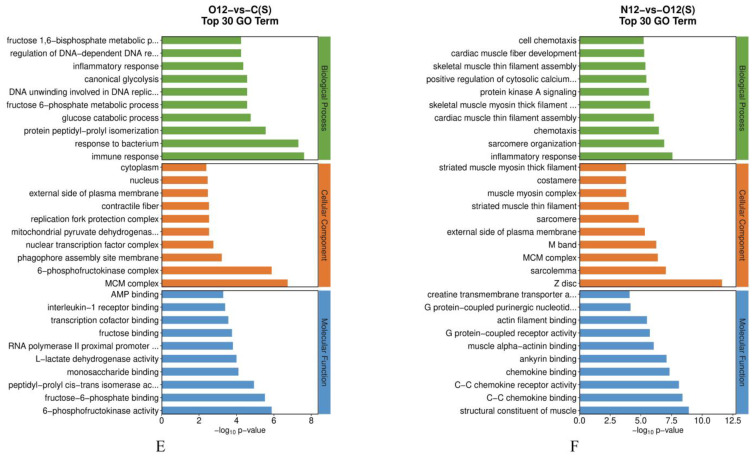
Illustration of the GO pathways in the different tissues under hypoxia and reoxygenation. (**A**–**C**) represent the results of pairwise comparisons between the brain tissues of groups C, N12, and O12, while (**D**–**F**) represent the results of pairwise comparisons between skin tissues of groups C, N12, and O12. The horizontal axes in the graphs display the names of the GO entries, and the vertical axes indicate the −log10 *p* values.

The GO enrichment analysis of the brain tissues (Figure 4A–C) revealed that, compared to control group C, group N12 exhibited an enrichment in its biological processes related to the response to mechanical stimuli. In contrast, group O12 was enriched in its biological processes related to hypoxia, sprouting angiogenesis, and its response to mechanical stress when compared to group C. Furthermore, the biological processes related to lysosomes and chemokines were enriched in group N12 compared to group O12. The GO enrichment analysis of the skin tissues (Figure 4D–F) showed that, in comparison to group C, group N12 displayed significant enrichments in the biological processes related to immune responses, chemotaxis, inflammatory responses, and leukocyte chemotaxis. The molecular functions related to chemokines were also enriched. Group O12, compared to group C, was enriched in its biological processes related to immune responses, responses to bacteria, and inflammatory responses. Group N12, compared to group O12, was particularly enriched in its inflammatory responses and molecular functions related to chemokines. A complete list of GO term names can be found in Table S1.

### 3.6. KEGG Enrichment Analysis

**Figure 5:**
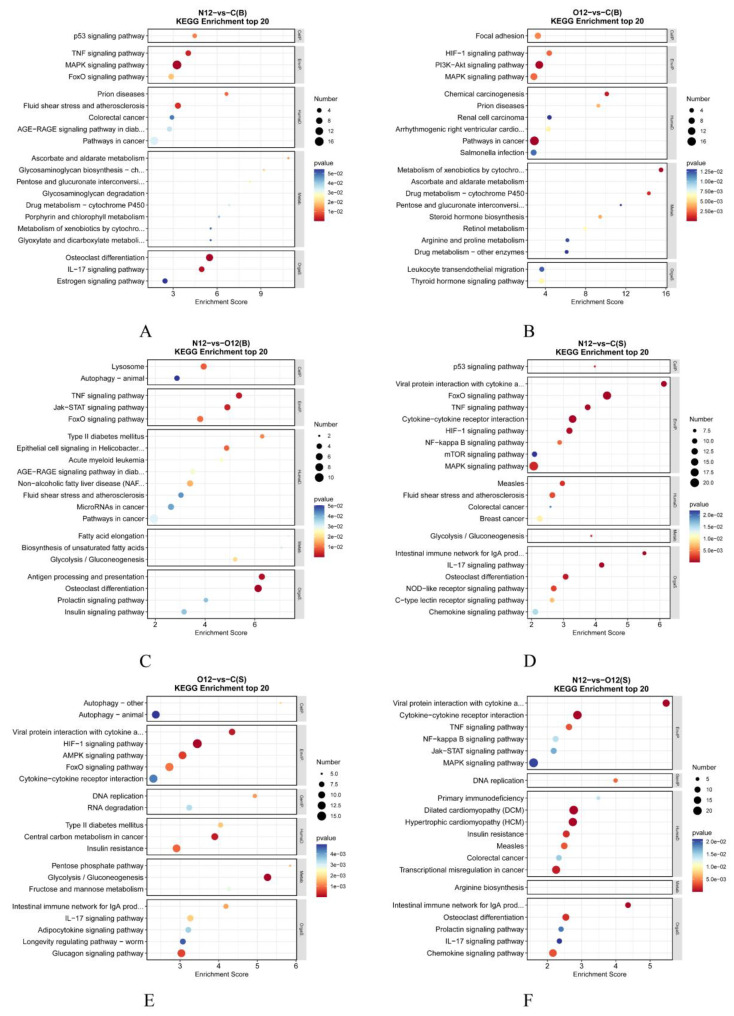
KEGG bubble plots representing the different tissues under hypoxia and reoxygenation. (**A**–**C**) present the results of two-by-two comparisons between the brain tissues of groups C, N12, and O12, while (**D**–**F**) show the outcomes of similar comparisons between the skin tissues of groups C, N12, and O12. The horizontal axes in the graphs represent the enrichment scores, where larger bubbles indicate higher numbers of differentially protein-coding genes within that entry. The bubble color transitions from blue to white and then to yellow and red with smaller enrichment *p*-values indicate greater statistical significance values.

The KEGG enrichment results for the brain tissues (Figure 5A–C) indicated that, compared to the control group C, group N12 showed enrichments in several immune-related pathways, such as the IL-17 and TNF signaling pathways. Additionally, the cytochrome P450-related pathways and estrogen pathways were enriched in group N12. Group O12 also exhibited enrichments in its cytochrome P450-related pathways, steroid hormone pathways, and thyroid hormone pathways. Furthermore, the PI3K-Akt signaling pathway was enriched in this set of comparisons. The HIF hypoxia pathway was also enriched in this set of comparisons. In the skin analysis, the KEGG enrichment analysis (Figure 5D–F) showed that the hypoxia-related pathway HIF, as well as several immune-related pathways and glycolytic pathways, were enriched in group N12 compared with the control group C. Group O12 was similarly enriched in numerous immune-related pathways and glycolytic pathways when compared to group C. Comparing the enriched pathways in the two tissues, it was observed that the skin tissues exhibited greater numbers of differentially enriched genes than the brain tissues. However, both tissues showed enrichments in the p53 pathways and the FoxO pathways. The complete KEGG term names can be found in Table S1.

### 3.7. Transcriptome Sequencing Validated Using qPCR

**Figure 6:**
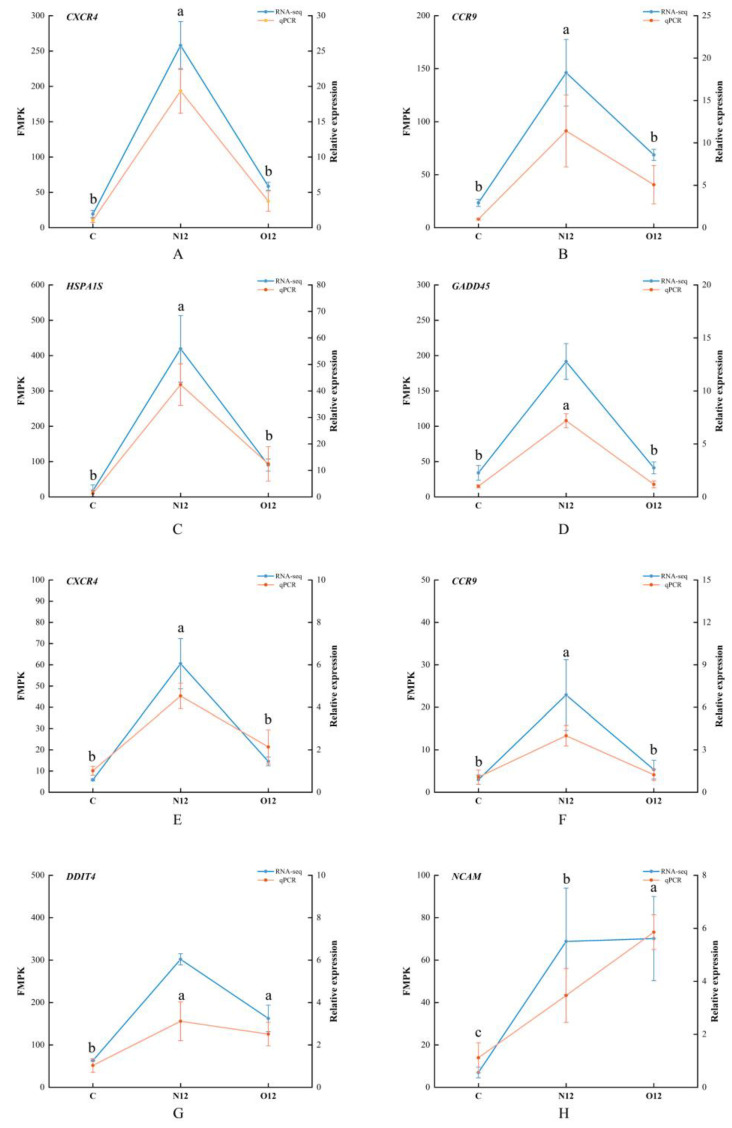
qPCR validation and RNA-seq data plots. The left *Y*-axes represent the gene expression levels in FMPK units obtained through the RNA-seq. The right *Y*-axes show the relative expression results obtained via qPCR. (**A**–**D**) correspond to the four validated genes in the skin tissues while (**E**–**H**) represent the four validated genes in the brain tissues. Different lowercase letters represent significant differences (*p* < 0.05), and the letters apply only to the comparison of qPCR data.

To validate the accuracy of the transcriptome sequencing, four genes from both the skin and brain tissues were chosen for qPCR verification. The gene expression patterns obtained through qPCR closely mirrored the FMPK values of the genes from the transcriptome sequencing (Figure 6). This alignment in expression trends reinforced the reliability of the sequencing results.

## 4. Discussion

In the mucosal immunity of fish, mucus cells play a crucial immunological role. The mucus produced by these cells serves as the first line of defense against external infections and damage [11]. In a hypoxia test conducted on seabass, the number of mucus cells in the skin was observed to increase. The experimental findings strongly suggested that mucous cell counts can serve as an indicator for monitoring the external stresses that fish encounter [29]. In this experiment, a significant increase in the number of mucous cells was observed in group N12 compared to group C, whereas no significant change in the number of mucous cells was observed in groups N3 and N6. This indicates that a short period of hypoxia stimulation could not lead to the proliferation of mucous cells in *P. ussuriensis*. As the duration of stress continued to increase, the skin of *P. ussuriensis* responded to the persistent external stress by actively increasing the number of mucus cells to enhance mucus secretion, while the number of mucus cells in group O12 was significantly lower than that in group C. That is, after a hypoxia–reoxygenation cycle (12 h hypoxia and 12 h reoxygenation), the mucus secretion capacity of *P. ussuriensis* was reduced. This suggests that 12 h of reoxygenation could not completely eliminate the negative effects of the same duration of hypoxia on the skin of *P. ussuriensis*.

In this experiment, the antioxidant system was activated in both tissues with increasing hypoxia time. The SOD activities in both tissues significantly rose and remained elevated even after reoxygenation. To counteract the accumulation of H_2_O_2_, CAT and GSH became effective. CAT activity in the skin progressively increased with the duration of hypoxia, and the GSH contents also significantly increased. Conversely, CAT activity in the brain did not exhibit significant changes, but the GSH contents in the brain tissues increased significantly, surpassing those in the skin. We postulated that this was because GSH plays a prominent role in scavenging H_2_O_2_ in the brain compared to CAT. The transient elevation of CAT activity in brain tissue in the N3 group followed by a return to normal levels also demonstrated that GSH was sufficient to scavenge H_2_O_2_. The changes in MDA also supported this hypothesis. The MDA levels in the brain tissues did not exhibit significant increases in any of the test groups, whereas the skin tissue MDA levels showed substantial increases. Cortisol, an essential stress hormone secreted by fish during stressful conditions, serves as a signaling molecule for the stress response in fish [30]. In a hypoxia study on Amur sturgeon (*Acipenser schrenckii*), there was a continuous increase in serum cortisol concentration [31], demonstrating that hypoxia stress leads to elevated cortisol levels. LZM levels rise in response to external threats when an organism is under stress. In this experiment, both the LZM and cortisol levels in the skin tissues showed significant increases with the increased duration of hypoxia. Conversely, the cortisol levels in the brain tissues exhibited smaller, though not statistically significant, increases with prolonged hypoxia. Similarly, the contents of LZM in the brain tissues did not show significant changes. This might be attributed to the fact that the brain, as a central nervous organ, is not directly involved in intense immune responses. The trends observed in this experiment were consistent with the results from an acute hypoxia stress test on juvenile hybrid yellow catfish (*Pelteobagrus fulvidraco × P. vachelli*), where the serum and liver tissue indices of SOD, CAT, LZM, MDA, and cortisol significantly increased during the pre-hypoxia phase [32]. The experimental findings confirmed that hypoxia stress triggers oxidative stress and immune responses in both the brains and skin of *P. ussuriensis*. However, the degree of oxidative stress and immune response in the brain, being a central nervous organ, is less pronounced than that in the skin.

This experiment analyzed and elucidated the transcriptome-level responses of both types of tissue to hypoxia stress. The skin exhibited a greater number of differential genes compared to the brain. In the brain, the GO enrichment analyses revealed that group N12 had enriched stress responses related to mechanical injuries compared to the control group. This demonstrated that hypoxia stress, as a physical pressure, activates the defense mechanisms in the brain of *P. ussuriensis.* Additionally, group O12 was directly enriched in its hypoxia-related pathways and vascular outgrowth proliferation pathways. This suggests that the effects of hypoxia on the brain do not completely disappear after reoxygenation. After reoxygenation, *P. ussuriensis* increased its oxygen acquisition capacity by actively increasing vascular neovascularization. In the skin, compared to the control group, group N12 exhibited direct enrichments in its immune-related pathways, including its inflammatory response, immune response, and chemotaxis. These immune and inflammatory responses remained enriched in group O12. Compared to the brain, the skin was directly enriched in its immune-related pathways. This was possibly due to the fact that the skin, as an organ in direct contact with the external environment, is more active and vigorous in its immune responses.

Specifically, in the KEGG enrichment analyses, when compared to the control group, the HIF pathways in brain tissues were enriched only in the O12 group after reoxygenation. In contrast, the HIF pathways in the skin tissues were enriched in both the N12 and O12 groups. The HIF pathway is an important regulatory pathway for the physiological response to hypoxia in vertebrates [33]. The difference in the time of appearance of HIF pathway enrichment in the two tissues may be due to the fact that the skin is in direct contact with the outside world and shows a more active self-protective response to hypoxia stress, and the brain is less affected than the skin tissue. The persistence of the HIF pathway after reoxygenation in both tissues provided evidence that the effects of acute hypoxia stress on fish are not completely eliminated after the same reoxygenation time. This was confirmed by the results of enzyme activity and hormone analyses. In terms of immunity, the number of immune pathways enriched in skin is greater than in brain and the types of immune pathways are mostly nonspecific, such as TNF, IL-17, and chemokine pathways. This suggests that fish immunity activated by hypoxia as a nonpathogenic stressor is predominantly nonspecific. The cytochrome P450 family plays a pivotal role in the oxidative transformation of endogenous and exogenous molecules. This role in response to hypoxia stress has was demonstrated in spotted seabass (*Lateolabrax maculatus*) during a hypoxia stress test [34]. In our experiment, the cytochrome P450-related pathway was enriched, further emphasizing the importance of the cytochrome P450 family in responding to hypoxia stress. This activation and enhancement of the immune response under hypoxia stress aligned with prior findings in studies on Ya fish (*S. prenanti*) [19], Amur sturgeon (*A. schrenckii*) [31], and large yellow croaker (*Larimichthys crocea*) [35]. The enrichment of the glycolytic pathway in both the brain and skin tissues suggested that *P. ussuriensis* adapts to hypoxia conditions by activating the glycolytic pathway to meet its energy requirements. A similar enrichment of the glycolytic pathway was observed in transcriptome analyses of the brain tissues of the silver carp (*Hypophthalmichthys molitrix*) following exposure to hypoxia stress [36]. Both the brain and skin tissues were enriched for the p53 pathway under hypoxia conditions. The p53 pathway can be activated by various stressors, including hypoxia stress. Stress signals are transmitted to the p53 protein through post-translational modifications, activating p53 as a transcription factor to initiate processes such as cell cycle arrest, cell senescence, or apoptosis [37]. This enrichment aligned with findings in the transcriptome analysis of goldfish (*Carassius auratus Linnaeus*) gills infected by *Myxobolus ampullicapsulatus*, where the role of p53 in triggering apoptosis in fish after infection was illustrated [38]. Furthermore, the FoxO pathway was enriched in both the brain and skin tissues under hypoxia stress. The FoxO pathway plays a crucial role in cell cycle control and the response to oxidative stress. In the muscle transcriptome analysis after hypoxia stress in blunt snout bream (*Megalobrama amblycephala*), the FoxO pathway, along with the p53 pathway, was also enriched, corroborating the importance of these pathways in the context of hypoxia stress [39]. Additionally, the PI3K-Akt signaling pathway, which was enriched in the brain tissues after reoxygenation, and the mTOR signaling pathway in the skin under hypoxia have been shown to play roles in fish immunity [40]. The MAPK pathway, which was also enriched in both tissues in response to hypoxia stress and its interaction with the HIF pathway, was confirmed to have similar trends in the bighead carp (*Hypophthalmichthys nobilis*) [41]. The role of hormones in fish immunomodulation is substantial. Besides hormones directly related to immunity, hormones such as prolactin, sex hormones, and others have been demonstrated to regulate immune responses in fish [42]. Pathways associated with steroids, prolactin, and estrogen were enriched in this experiment. We hypothesize that the hormones associated with the above pathways may play a role in the response of *P. ussuriensis* to hypoxia stress or that their secretion is affected by hypoxia. The mechanisms by which different hormones regulate the recovery of fish in response to hypoxia stress and after reoxygenation still need to be further investigated. In conclusion, KEGG pathway analyses showed that 12 h of hypoxia activated the hypoxia regulatory and nonspecific immune system of *P. ussuriensis*. The organism internally replenishes energy supply through glycolysis and reduces the damage caused by hypoxia by regulating the cell cycle. The same reoxygenation time did not completely alleviate the negative effects of hypoxia.

## 5. Conclusions

The present study provided insights into the oxidative stress and immune responses of *P. ussuriensis* under acute hypoxia stress by analyzing its histophysiology and transcriptomics. The results indicated that acute hypoxia and subsequent reoxygenation led to changes in the number of mucous cells in the skin of *P. ussuriensis*. This suggested that mucous cell count can serve as an indicator for monitoring the stress-induced immune status of *P. ussuriensis*. Hypoxia induced oxidative stress and immune responses in the fish, and the adverse effects of hypoxia stress persisted even after reoxygenation treatment within the same time frame. A comparison between the two tissues revealed that the overall response in the brain was less pronounced than that in the skin. This study enhances our understanding of the immune response in the brain and skin of *P. ussuriensis* under conditions of acute hypoxia and subsequent reoxygenation.

## Figures and Tables

**Table 1:** Primer sequences.

Gene Name	Sequences (5′-3′)	Product Length (bp)
*β-actin*	F: AGAGCGTAACCCTCGTAG	235
	R: CTGCTTTGCGGCTGAATA	
*CXCR4*	F: GCCGTTCTATGCCGTGGATG	107
	R: GGATGAGGACGCTGCTATACAAG	
*CCR9*	F: GTACCTGCTGAACCTTGCCTTAG	121
	R: CAACGCCGATGTGCCCTTAC	
*HSPA1S*	F: TTACGGTGCGGCGGTTCAG	80
	R: CCACATCCAAGAGCAGCAAGTC	
*DDIT4*	F: GAGTGAGAGTGTTTGGGCTGATG	137
	R: CAGAACCAGTATCGGAGCAATCG	
*NCAM*	F: TGGTGAGAATGCGAAGGTTGTG	124
	R: GTAGCGAGAGGAATCTGATGTGTC	
*GADD45*	F: GCTGCGAGAACGACATCAACATC	83
	R: CTCCTTGGTGCTTGGCTCTCC	

**Table 2:** Number of mucus cells.

	C	N3	N6	N12	O12
Number of mucus cells	29.67 ± 3.30 ^b^	25.33 ± 3.68 ^b^	32.33 ± 2.36 ^b^	49.67 ± 5.19 ^a^	13.67 ± 2.05 ^c^

The number of mucus cells was counted in three randomly selected fields of view in each section. Values are means ± SD of three replicates. The different lowercase letters indicate significant differences at *p* < 0.05.

**Table 3:** Results of the sequencing data quality.

Sample	Raw Reads (M)	Raw Bases (G)	Clean Reads (M)	Clean Bases (G)	Valid Bases (%)	Q30 (%)	GC (%)
C-B1	48.21	7.23	47.6	6.91	95.59	93.41	46.1
C-B2	51.69	7.75	51.05	7.42	95.67	93.62	46.02
C-B3	49.6	7.44	48.99	7.12	95.68	93.32	45.93
C-S1	49.19	7.38	48.59	7.05	95.49	94.39	47.52
C-S2	48.25	7.24	47.65	6.9	95.35	94.43	47.45
C-S3	49.09	7.36	48.45	7.02	95.32	93.74	47.46
N12-B1	51.04	7.66	50.47	7.32	95.65	93.37	45.99
N12-B2	51.05	7.66	50.44	7.35	95.95	94.03	46.11
N12-B3	48.77	7.32	48.07	6.98	95.35	93.96	46.01
N12-S1	51.75	7.76	51.1	7.42	95.59	93.7	47.53
N12-S2	48.2	7.23	47.57	6.91	95.62	93.76	47.62
N12-S3	48.22	7.23	47.63	6.93	95.78	93.61	47.44
O12-B1	48.09	7.21	47.36	6.84	94.86	94.06	45.92
O12-B2	50.49	7.57	49.75	7.18	94.75	93.97	46.05
O12-B3	48.91	7.34	48.26	7.01	95.58	93.99	45.92
O12-S1	47.81	7.17	47.09	6.80	94.89	94.29	47.28
O12-S2	47.36	7.10	46.67	6.74	94.80	94.24	47.26
O12-S3	47.21	7.08	46.39	6.68	94.31	94.42	47.39

## Data Availability

The data presented in this study are available in this article.

## Supplementary Materials

The following supporting information can be downloaded at: https://www.mdpi.com/article/10.3390/ani14020246/s1, Table S1: Complete GO and KEGG term names.
